# Molecular Mechanism and Role of Japanese Encephalitis Virus Infection in Central Nervous System-Mediated Diseases

**DOI:** 10.3390/v14122686

**Published:** 2022-11-30

**Authors:** Pardeep Yadav, Pratik Chakraborty, Niraj Kumar Jha, Saikat Dewanjee, Abhimanyu Kumar Jha, Siva Prasad Panda, Prabhu Chandra Mishra, Abhijit Dey, Saurabh Kumar Jha

**Affiliations:** 1 Department of Biotechnology, School of Engineering & Technology (SET), Sharda University, Room # 311, Block-01, Plot No. 32–34, Knowledge Park III, Greater Noida 201310, India; 2 Advanced Pharmacognosy Research Laboratory, Department of Pharmaceutical Technology, Jadavpur University, Kolkata 700032, India; 3 Pharmacology Research Division, Institute of Pharmaceutical Research, GLA University, Mathura 281406, India; 4 Department of Life Sciences, Presidency University, Kolkata 700073, India; 5 Department of Biotechnology Engineering and Food Technology, Chandigarh University, Mohali 140413, India; 6 Department of Biotechnology, School of Applied & Life Sciences (SALS), Uttaranchal University, Dehradun 248007, India

**Keywords:** autoimmune encephalitis, BBB permeability, CNS, Japanese encephalitis virus, neurodegeneration, proinflammatory mediators

## Abstract

The Japanese encephalitis virus (JEV) is the most common cause of neurodegenerative disease in Southeast Asia and the Western Pacific region; approximately 1.15 billion people are at risk, and thousands suffer from permanent neurological disorders across Asian countries, with 10–15 thousand people dying each year. JEV crosses the blood-brain barrier (BBB) and forms a complex with receptors on the surface of neurons. GRP78, Src, TLR7, caveolin-1, and dopamine receptor D2 are involved in JEV binding and entry into the neurons, and these receptors also play a role in carcinogenic activity in cells. JEV binds to GRP78, a member of the HSP70 overexpressed on malignant cells to enter neurons, indicating a higher chance of JEV infection in cancer patients. However, JEV enters human brain microvascular endothelial cells via an endocytic pathway mediated by caveolae and the ezrin protein and also targets dopamine-rich areas for infection of the midbrain via altering dopamine levels. In addition, JEV complexed with CLEC5A receptor of macrophage cells is involved in the breakdown of the BBB and central nervous system (CNS) inflammation. CLEC5A-mediated infection is also responsible for the influx of cytokines into the CNS. In this review, we discuss the neuronal and macrophage surface receptors involved in neuronal death.

## 1. Introduction

Japanese encephalitis virus (JEV) is a zoonotic, neurotropic virus belonging to the family Flaviviridae. Japanese encephalitis (JE) is characterized by capacious inflammation of the central nervous system (CNS) followed by the disruption of the blood-brain barrier (BBB). JE was first recognized in horses and humans back in the 19th century. A high rate of mortality and severe psychiatric and neurological sequelae among the surviving population contribute to the dreadfulness of the disease. In South-East Asia and Western Pacific countries, JE-mediated fatalities are a matter of serious concern regarding public health [1]. JEV is transmitted in humans mainly through *Culex* mosquitoes. Interestingly, the direct human-to-human transmission does not take place, while many domestic animals and migratory birds play as host pools for JEV. The possibility of JEV spreading to new geographical locations is immensely high with the increasing mosquito habitat as a result of global warming.

JEV is an enveloped virus containing ssRNA as its genomic material. The genome codes for a single polyprotein that cleaves into three structural proteins. They, in turn, act as precursors for membrane proteins and seven non-structural proteins (NSPs). The E protein is the main target to neutralize antibodies, containing the cellular receptor binding sites and the fusion peptide. Neuroinflammation, vasculitis, and neuronal degeneration are commonly associated with JEV infection [2]. Occludin, a key member of the tight junction complex of BBB, is very sensitive to inflammatory modulations and oxidative stress [3]. JEV also affects brain microvascular endothelial cells (BMECs), indirectly influencing the downregulation of expressions of claudin 3 and 5. JEV can replicate within glial cells, facilitating the release of soluble mediators that add to the damage to claudin 5 and zonula occludens 1. Proinflammatory cytokines and/or chemokines also play significant roles in altering BBB permeability during JE. Release of interleukin 6 (IL-6), vascular endothelial growth factor (VEGF), and matrix metalloproteinases (MMPs) from astrocytes and pericytes are triggered by JEV infection [4]. Clinically, JE-mediated mortalities can be linked with high viral titers within the brain, elevated levels of inflammatory cytokines and chemokines, and disruption of BBB [5]. However, the scientific community is yet to arrive at a unanimous decision on whether BBB disruption is a prerequisite or a consequence of JEV infection. The present article aims to evaluate and summarize the molecular mechanisms involved with JEV infection, and its influence on CNS abnormalities, with an emphasis on neuronal and macrophage surface receptors involved in neuronal death.

## 2. Epidemiology

In the 1870s, the first JE patient displayed neurological impairments as a result of a flavivirus infection, which belongs to the Flaviviridae family [6,7]. The patient’s brain sample exhibited neuronal degeneration and inflammation in the CNS. The JEV is predominantly transmitted by the *Culex* mosquitoes, mainly *C. tritaeniorhynchus*, *C. vishnui*, and *C. Seudovishnui*, while *Anopheles subpictus* is a secondary vector for this virus [8]. The majority of transmission to humans is thought to occur in places where domestic animals act as reservoirs or amplifying hosts, and some migratory bird species have been identified as reservoir hosts for the JEV [9]. Pigs are good zoonotic hosts, while cattle and horses are thought to be the dead-end hosts for JEV. JEV has a significant fatality rate and affects more than 24 nations in South-East Asia and the Western Pacific region [6]. JE affects more than 50,000–75,000 people globally, 30–50% of whom develop permanent neurological abnormalities, and about 10,000–15,000 people die from the disease per year [10,11]. Neurological disorders occur mainly due to the invasion of JEV into the CNS by crossing the BBB [9,12]. India is the nation that is most impacted by the JE, with a high fatality rate [13]. JEV was identified in India in the 1950s through a serological survey, and the first outbreaks were detected in West Bengal in 1973 [14]. In Northern India in 1978, Gorakhpur was the area that was most impacted by the JE [15]. Since 2006, three vaccination campaigns have been implemented in the epidemic zones of India: two use the inactivated Vero cell-derived SA 14-14-2 and d 821564XY JE vaccines, and one uses the live attenuated LAV-SA 14-14-2 vaccine [13,16,17,18]. Between 2005 and 2010, 205 deaths were recorded, with 2040 active cases of JE in Nepal [19]. Since the Terai district of Nepal was more severely affected than the rest of the nation, the government of Nepal began widespread vaccination efforts with a live attenuated virus (LAV-SA 14-14-2) in Terai provinces in 2001 [13]. Based on the effectiveness of the immunization programs, scientists have divided 27 nations in the world into eight groups (Table 1). Furthermore, the governments of a few out of these 27 nations operate programs at various levels to lower the force of infection (FOI) of JEV. Some are successful in reducing FOI by giving proper vaccination and treatments (Table 2).

## 3. Pathophysiology of JE in CNS

The breaching of BBB is one of the most distinctive pathophysiologies of JEV. In addition to causing neuronal cell death, JEV propagation in astrocytes and microglia causes an upregulation of proinflammatory cytokines. JE is directly related to the inflammation and vasculitis of the brain and is associated with the invasion of the virus into the brain. JEV can infect BMECs, astrocytes, microglia, and pericytes, among other cell types found in the BBB. However, BMEC infection does not affect cell viability proposing that the BBB penetration by the JEV is not associated with JEV-mediated cell death. JEV infection actually suppresses tight junction protein expression and alters adherent localization, thus disrupting the tight junctions between the BMECs [46,47,48]. JEV can replicate in astrocytes and pericytes and simultaneously endorse soluble mediator release that damages tight junction proteins, especially zonula occludens 1 and claudin 5. JEV infection-mediated release of IL-6, VEGF, MMP2, and MMP9 triggers astrocytes and pericytes, playing key roles in enhancing endothelial permeability [48]. JEV infection also causes microglial activation resulting in the release of tumor necrosis factor α (TNF-α), IL-1β, IL-6, monocyte chemoattractant protein-1 (MCP-1), chemokine (C-C motif) ligand 5 (CCL5), CXC motif chemokine ligand 10 (CXCL10), and inducible nitric oxide synthase, which may be related to endothelial barrier damage [46,47]. The breakdown of the BBB caused by JEV infection appears to be more of a side effect than a direct cause of viral proliferation in BMECs [49]. Thus, elevated levels of inflammatory cytokines and chemokines, high viral titers in the brain, and fatalities caused by JE can all be connected.

JEV infection increases the production of proinflammatory cytokines, chemokines and signal transducers associated with the interferon γ (IFN-γ) pathways [46]. Factors responsible for the analog of the JEV infection in the CNS are poorly understood. Mice affected with JEV show a high amount of cytokine and chemokine production in the brain [50]. However, JEV immune splenocyte transfer could protect mice from extraneural JEV infection [50]. Profiling of genes using high throughput screening (HTS) authorizes the identification of the critical genes that play an important role in the JEV infection and modulation of the pathways and also reveals the cellular and molecular pathways associated with this infection [51,52]. Twenty-three out of 173 genes of human glioblastoma cells infected by the West Nile virus (another flavivirus) have been identified to play important roles in cellular neurodegeneration via microarray analysis [53]. Thus, indications are quite clear that flavivirus infection does lead to neurodegeneration. Neurovirulence genes from West Nile virus-infected mice were found to be engaged in a variety of signaling cascades, including protein degradation, T cell and MHC class I and II antigen presentation, and apoptosis [54]. Apoptosis, triggered during the replication of JEV, leads to the death of neuronal and non-neuronal cells [55]. In human and mouse neuroblastoma cells, the energizing of tumor necrosis factor 1 (TNFR1) and signaling through tumor necrosis factor associated death domain (TRADD) prompt downstream apoptotic cascades during JEV infection [55]. Lipocalin ApoD, which is associated with brain injury and is also expressed in response to JEV, was one of the CNS-specific proteins discovered to have increased expression in mice brains infected with West Nile virus strains.

Dimeric NS1 is a multifunctional glycoprotein that participates in JEV replication’s complex building and replication by interacting with other JEV non-structural proteins as well as many host proteins such as RPL18, RPL18a, vimentin, and hnRNP K. In contrast, it appears that the hexameric form of NSP1 primarily modulates the host immune system in order to favor JEV replication [56]. Furthermore, an extended variant of the NS1 protein, NS1′ (~53 kDa), has been shown to decrease IFN type I activity, a critical component of the host’s innate and adaptive immune response to viral infections [57]. NS2A is necessary for virion assembly in addition to replication because it transports the newly synthesized (+) sense single-stranded RNA from the replication complex to the assembly complex. It has also been shown that NS2A plays an important role in JEV infection by reducing host cell antiviral responses, where NS2A decreases protein kinase-induced cell death [58]. JEV relies on NS1′ to survive inside the host cell via modulating the host immune response, and a single mutation in NS2A precludes NS1′ production [59]. As a result, as a regulator of NS1′, the NS2A protein is critical in JEV infection and pathogenesis. In a contrasting development, experimental techniques based on reverse genetics developed a synthesis route for NS1′ via A66G alteration in the NS2A gene of the JEV SA14-14-2 strain contributed to the recovery of the GC-rich pseudoknot and the creation of the NS1′, which in turn may potentially act as a biomarker for virulent virus infection [60]. By causing the AXL membrane protein to be degraded by the ubiquitin-proteasome pathway, the NS2B-NS3 protein complex induces cellular apoptosis. Since they are essential for JEV replication and post-translational processing of the polyprotein, NS2B and NS3 are suitable therapeutic targets [61]. NS5 is essential for JEV replication because of the polymerase activity of its RNA-dependent-RNA-polymerase (RdRp) domain and the 5′-capping enzymatic activity of its methyltransferase domain. There are three subdomains of the NS5 RdRp domain: palm, thumb, and finger. The palm subdomain, which is composed of conserved aspartic acid residues, serves as the active site for the binding of RNA, metal ions, nucleotides, and other molecules, as well as for the transfer of phosphate groups. The finger subdomain creates a tunnel to guide the template RNA to the active site, while the thumb subdomain is necessary to assemble an RNA synthesis complex and control RNA synthesis. NS5 is a potential target for the creation of therapeutic moieties due to its crucial function in JEV replication. JEV infection also triggers intracellular Ca^2+^ overload, which in turn correlates with abnormalities of mitochondrial membrane potential and protein kinase B (Akt)/mammalian target of rapamycin (mTOR) and Janus tyrosine kinase (JAK)/signal transducer and activator of transcription 1 (STAT1) signaling pathways. Ubiquitin also plays a vital role in that fundamental cellular functions like endocytosis, protein breakdown, and immunological signaling are regulated by ubiquitin.

## 4. Overview of the JEV Genome

The linear (+) ssRNA genome of the JEV, an enveloped virus, is surrounded by several copies of the capsid protein. The viral nucleic acid and capsid protein combine to produce a nucleocapsid enveloped by a lipid bilayer obtained from the host. The genomic RNA has a methylation cap at its 5′ end but no poly-A tail at its 3′ end. A single open reading frame (ORF) is sandwiched between two brief non-coding regions (NCR) at the 5′ and 3′ ends of the genomic RNA, which is around 11 kb in length. The ORF encodes a 3400 amino acid polyprotein that is cleaved by both viral and host proteases to generate three proteins, viz. the C protein, the M/prM protein, and the E protein and seven NSPs, i.e., NS1, NS2A, NS2B, NS3, NS4A, NS4B, and NS5 [62]. All mosquito-borne flaviviruses include highly conserved NCRs, which create secondary structures to facilitate viral replication, transcription, and translation. The 5′ NCR contains functional RNA components like promoters, enhancers, and potential cyclization sequences essential for the interaction between the genomic RNA’s distantly placed 5′ and 3′ NCRs [63]. During viral infection, the NS5 protein binds with circularized RNA and begins RNA replication at the 3′-NCR. The JEV NS2B-NS3 protease is notable for performing proteolytic cleavages between NS2A and NS2B, NS2B and NS3, NS3 and NS4A, and NS4B and NS5. Proteolytic cleavages between C and prM, prM and E, E and NS1, and NS4A and NS4B are carried out by the host protease signalase in the polyprotein. The N-terminal region of the JEV NS3 protein, which also functions as a binding site for the cofactor protein NS2B, contains protease activity. Additionally, the helicase activity of NS3’s C-terminal domain causes the double-stranded RNA to unwind negatively during viral RNA replication. Because its N-terminal domain (methylase domain) has methylase activity needed for the 5′ capping of naive viral RNA and its C-terminal domain (RdRp), NS5 is also a crucial protein.

The five genetically distinct lineages of the Japanese encephalitis virus, which diverged in the sequence of GV, GIV, GIII, GII, and GI, have evolved in nature. By nucleotide sequencing of the C/PrM and E genes, five JEV genotypes have been identified. The earliest JEV lineage is formed by genotypes IV and V, while genotypes I, II, and III are the most common, accounting for 98% of the strains isolated between 1935 and 2009 [10,64]. Mutations in NS2B/NS3 increase the infectivity of GI JEV in amplifying hosts. JEV GI has supplanted GIII as the prevalent genotype in typical Asian epidemic locations, but GIII has moved from Asia to Europe and Africa, causing domestic JE cases in Africa. GII and GV, which were endemic in Malaysia, also showed significant geographical shifts. GII traveled southward, resulting in the frequency of JE in Australia, while GV resurfaced after decades of silence in China and the Republic of Korea. Along with these developments, JE emerged as an emergent infectious disease in certain non-traditional epidemic areas. JEV regional changes represent a significant hazard to human health, resulting in massive disease burdens.

## 5. Molecular Targets Associated with JEV Infection

**Figure 1:**
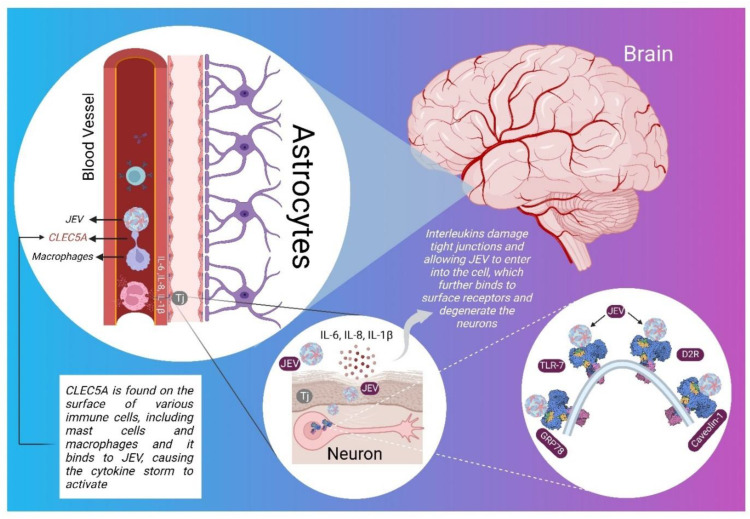
Interleukins (IL-6, Il-8, and IL-1) breakdown tight junction existing between endothelial cells of the blood-brain barrier of JEV-infected patients and target proteins present on the surface of ER of neurons and other surfaces of neurons, as shown in a systematic figure.

JEV is a flavivirus with a genome of about 11 kb ssRNA. Cell type-specific interactions of viral proteins with host machinery critically influence the nature of pathogenic outbursts. NS5 augments proinflammatory responses by disrupting host lipid metabolism, leading to elevated neurovirulence and neuroinvasiveness [65]. Researchers have identified (Figure 1) a few surface receptors mainly responsible for the entry of JEV into the nerve cells, namely C-type lectin domain family 5 member A (CLEC5A), Glucose regulated protein 78 (GRP78), Caveolin-1, D2-receptor (D2R), toll-like receptors (TLRs), and Src protein [46].

### 5.1. CLEC5A

CLEC5A, present on the surface of myeloid cells, monocytes, macrophages, osteoclasts, dendritic cells, and neutrophils, is an integral membrane protein without any signaling motif, with a small cytosolic end (tail) justifying the requirement of an adapter protein DAP12 for exerting physiological activity [66,67]. DAP12 completes the signaling for CLEC5A via spleen tyrosine kinase SYK (SYK pathway) [68]. Numerous cytokines, e.g., TNF-α, IL-1, IL-6, IL-8 and IL-17A, and chemokines like MIP-1, RANTES, CXCL10 and macrophage-derived chemokine are produced when CLEC5A is activated. Additionally, it increases innate immune response [68,69]. CLEC5A acts as the gateway for the entry of many viruses like Dengue virus, Influenza virus (H5N1), and JEV within the cell [70]. The inhibition of CLEC5A prevents autoimmune inflammation, attenuating proinflammatory cytokines and suppressing cell infiltration in joints [66,67]. JEV directly interacts with CLEC5A and promotes DAP12 phosphorylation in macrophages. JEV stimulates macrophages, causing them to release proinflammatory cytokines and chemokines, which are significantly reduced in CLEC5A −/− macrophages infected with JEV.

### 5.2. GRP78

GRP78 is a member of the HSP70 family found on the surface of the endoplasmic reticulum (ER) of all nucleated cells [71]. GRP78 consists of 654 amino acids, responsible for accurate protein folding and assembly and inhibition of transport of misfolded ones [72]. In the ER lumen, GRP78 is well recognized for attaching to hydrophobic patches on nascent polypeptides and for its role in signaling the unfolded protein [73]. Out of the two domains of GRP78, the amino-terminal contains the ATP binding domain (ABD) and/or nucleotide-binding domain (NBD), and the carboxyl-terminal contains the substrate binding domain (SBD) [72]. JEV binds to GRP78 to gain entry into the host cell, whereby the recombinant JEV envelope protein domain III interacts with GRP78. GRP78 is overexpressed on the surface of cancerous cells, enhancing the chances of JEV infection in cancer patients. Antibodies against GRP78 greatly reduced the entry of JEV into cells [74]. Small interfering RNA (siRNA)-mediated GRP78 depletion markedly inhibited JEV entry to mouse Neuro2a cells [75].

### 5.3. Caveolin-1

JEV penetrates primary human BMECs via an endocytic route mediated by caveolae [76]. Caveolin-1 contains 82–101 amino acids with an α-helix juxtaposed to the membrane. Caveolin-1 has two primary functional sites, i.e., a scaffolding domain and tyrosine 14 (Y14) [77]. Another protein, ezrin, is a host protein essential for the caveolae-mediated endocytic pathway. In one study, scientists observed that ezrin-mediated actin cytoskeleton polymerization is essential for JEV internalization in human BMECs [76]. JEV enters human neuronal cells through caveolin-1-mediated endocytosis, which is dependent on the two-step regulation of actin cytoskeleton remodeling triggered by Rac1 and RhoA [78]. Rac1 activation first aided in caveolin-associated viral internalization besides promoting the phosphorylation of caveolin-1. RhoA is activated specifically as a result of virus attachment via activating the EGFR-PI3K signaling pathway. JEV infection is inhibited by siRNAs targeting caveolin-1, a key component of caveolae membranes involved in receptor-independent endocytosis. Herein, other targets for JEV inhibition include genes encoding the endosomal sorting complex required for transport (ESCRT) machinery vacuolar protein sorting 4 homolog A (VPS4A) and hepatocyte growth factor-regulated tyrosine kinase substrate (HGS), membrane fusion genes synaptotagmin 1 and N-ethylmaleimide sensitive factor, vesicle fusing ATPase (for N-methylmaleimide sensitive factor), actin polymerization genes VIL2, ACTR2, ACTR3, ARPC3, ARPC4, RAC1 and WASp actin nucleation promoting factor, and vesicle and endosomal transport genes COPA, GAF1, RAB4B, RAB5A, RAB5B and RAB11B [76].

### 5.4. D2R

**Figure 2:**
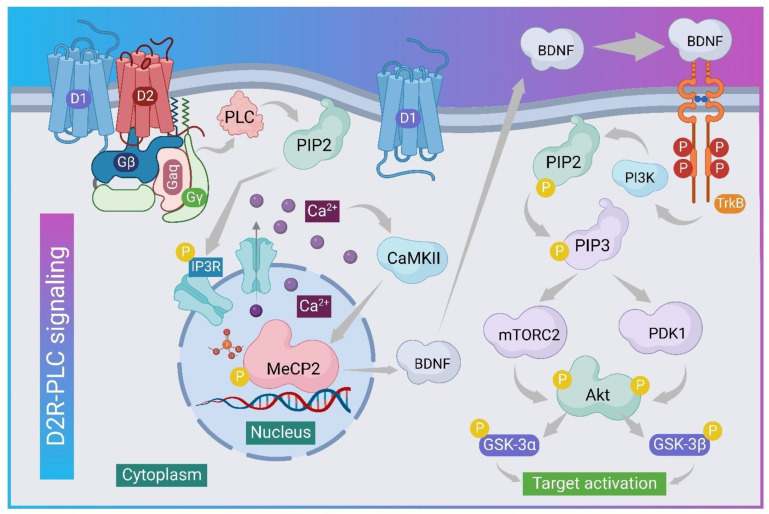
The graphic depicts the phosphorylation of several proteins at various levels of signaling to activate BDNF from MeCP2 inside the nucleus to activate the TrkB receptor for the activation of PIP3 to mTORC2 and PDK1 proteins, followed by Gsk to reach the target for function.

JEV specifically targets brain areas rich in dopaminergic neurons, such as the thalamus and midbrain [79]. Evidence suggests that JEV utilizes dopaminergic signaling to promote infection via modulating dopamine levels [80]. Dopaminergic D2R has a greater affinity for dopamine than the D1 receptor (D1R) [81]. D2R agonists boost JEV infection by activating phospholipase C (PLC) to increase surface expression via integrin β-3 and the upregulation of vimentin [79]. Dopamine, on the other hand, is the primary catecholamine neurotransmitter that regulates a wide range of biological functions such as cognition, endocrine regulation, and voluntary movement. Furthermore, the phosphorylation of tyrosine hydroxylase (TH) regulates dopamine biosynthesis and converts tyrosine to L-DOPA [82]. Experimentally, BE(2)-C neuroblastoma cells with high TH activity for dopamine synthesis were selected to investigate the level of dopamine during JEV binding to D2R [83,84]. The results showed that JEV modulates the level of dopamine during infection, with higher secretion in the early hours (3–6 h post-infection) and lower secretion in the late hours (24–36 h post-infection). In addition, D2R antagonists (prochlorperazine, haloperidol, and risperidone) were found to reduce JEV-NS3 protein expression [85]. JEV infection and JEV-NS3 synthesis are accelerated by activating D2R (agonist: quinpirole hydrochloride) [79,86]. Furthermore, PLC activation by D2R stimulation via Gq hydrolyzes PIP2 to IP3 and is responsible for the activation of Ca^2+^ release from intracellular reserves (Figure 2). According to research findings, the levels of cAMP in mock and JEV-infected BE(2)-C cells were comparable, but the PLC non-cytotoxic inhibitor inhibited JEV-NS3 expression as a result of targeting D2R-PLC signaling [87,88].

### 5.5. TLRs

It has been observed that viral replication occurs within astrocytes and microglia cells, producing proinflammatory cytokines that induce indirect neuronal death [89,90]. JEV-specific T cells and virus-neutralizing IgG and IgM antibodies play an important role in viral clearance from the CNS and peripheral lymphoid organs [91]. Neurons and other CNS cells actively respond to viral infection by producing type I IFN [92]. There is also growing evidence that the type 1 IFN pathway can be triggered by recognizing TLRs. Retinoic acid-inducible gene I and melanoma differentiation-associated protein 5 detect RNA in the cytosol and send signals via the adaptor protein mitochondrial antiviral-signaling protein [93]. TLRs that are surface-bound or endosomal identify ssRNA, dsRNA, and signals via myeloid differentiation primary response 88 (Myd88) and Toll-interleukin receptor-domain-containing adapter-inducing interferon-β (TRIF) molecules, showing that TLR signaling is involved in flavivirus-mediated immune responses. Following JEV infection, TLR4 and TLR3 play distinct signaling roles [94,95,96]. TLR3-deficient mice were extremely vulnerable to JE [90]. TLR3−/− mice had higher levels of proinflammatory cytokines and BBB permeability. TLR4 knockout mice showed increased resistance to JEV infection [97]. The loss of TLR4 caused powerful type I IFN responses in dendritic cells and macrophages via greater stimulation of antiviral IFN-stimulated genes via distinct activation of IFN regulating factor 3 and nuclear factor kappa-light-chain-enhancer of activated B cells (NF-κB) [97]. TLR7 and TLR8 identify ssRNA, and the recognition may vary depending on the species. TLR7 and TLR8 in mice and humans identify SS GU-rich RNA as a natural ligand. The stimulation of TLR7 and TLR8 activates TRIF and MyD88-dependent signaling pathways, which in turn stimulate the production of proinflammatory cytokines, chemokines, and type 1 IFNs [98]. TLR8 overexpression in TLR7−/− mice has no effect on mice survival as TLR8 compensates for another TLR [99]. The antiviral and anticancer activity was demonstrated by targeting the TLR-MyD88 dependent pathway, which produces the proinflammatory cytokine IFN and TLR7 agonist has antitumor action in cell line [98].

### 5.6. Src Protein

Tyrosine-protein kinase proto-oncogene Src is a non-receptor tyrosine kinase protein encoded by the SRC gene. It is a 60 kD phosphoprotein having around 533 amino acid residues, transmitting signals to control a multitude of cellular functions, including proliferation, differentiation, motility, and adhesion. Researchers suggest that NS5 and NS3 can be phosphorylated and associated with the Src family kinase activity [100]. In response to JEV infection, microglia releases proinflammatory cytokines via the Src/Ras/extracellular signal-regulated kinase (ERK) pathway [100]. On infection with JEV, the synthesis of Src also increases, which autophosphorylates itself and other signaling proteins such as rat sarcoma (Ras), rapidly accelerated fibrosarcoma (Raf), and ERK [101]. Protein tyrosine phosphorylation events play important roles in physiological signaling processes such as inflammation. Both inhibitors of the Src family, protein tyrosine kinase (PTK) and Ras, are found to inhibit JEV-induced ERK activation [100]. Inhibitors of PTK, Ras, and ERK substantially inhibited JEV-induced proinflammatory cytokine production and neurotoxicity. Caveolin-1 could be phosphorylated by Src in the presence of ezrin, which in turn mediates actin rearrangement as a crucial step for JEV entry [76]. The Src protein is a part of the tyrosine phosphorylation pathway, which plays an important role in the JEV-induced expressions of proinflammatory cytokines. During early JEV infection, lipid rafts operate as signaling platforms for Src tyrosine kinases, resulting in phosphoinositide 3′-kinase/Akt signaling activation [102]. These lipid rafts support the linking bridge in transducing TNF-α and IL-1β. JEV activates the Src/Ras/Raf/ERK/NF-κB signaling axis in the neuron/glial co-culture system in a reactive oxygen species (ROS)-dependent manner [61]. ROS and K^+^ efflux are found to induce NLRP3 inflammasome signaling in JEV-infected cultured mouse microglia and mouse brains, leading to the development of an inflammasome complex, caspase-1 activation, and the synthesis of mature cytokines [103]. The neurotoxic microglia activating phenotype and subsequent inflammatory responses are strongly linked with C-C chemokine receptor type 2 (CCR2) expression generated by JEV on the surface of microglia [59]. As a result of this Src protein’s role in numerous cellular processes, we can create an antiviral treatment strategy by possibly suppressing it [104,105].

## 6. Autoimmunity in JEV Infection

Although the specific mechanism of neuronal death in JE is yet to be clearly disclosed, studies suggest that MMPs have a role in neuronal cell death [106,107]. A study suggested that JEV infection increases the expression of MMPs (MMP2, 7, and 9) and TIMPs (TIMP1 and 3) and may contribute to the severity of the disease [106]. Evidence also shows that MMP9 expression is elevated during JEV infection in rat brain astrocytes via the formation of ROS during JEV infection [106]. MMP2, TIMP2, and TIMP3 concentrations were found to be higher in the CSF and serum of JEV-infected children compared to controls [107,108]. Furthermore, increased blood concentrations of MMP9 and MMP7 have been observed in JEV patients when compared to healthy controls. MMPs are also associated with glaucoma affecting the optic nerve, retinal ganglionic cells and connected structures for vision [109,110]. Another autoimmune condition that affects youngsters is autoimmune encephalitis. Following JEV infection of the CNS, antigen exposure may cause autoimmune encephalitis, and deep cervical lymph nodes are emptied of neuronal surface antigens, activating antigen-specific memory B lymphocytes and stimulating the formation of autoantibodies [111]. However, memory B cells can also go to the brain and be restimulated, where they undergo antigen-driven affinity maturation, clonal expansion, and differentiation into antibody-producing plasma cells [110,112]. In a study, researchers found that the CSF of patients was negative for JEV RNA but positive for anti-neuronal surface antibodies, anti-NMDAR antibodies and anti-γ-aminobutyric acid B receptor antibodies, and there were no significant differences between the patients who acquired autoimmune encephalitis in terms of gender, CSF protein level, CSF WBC count, complications, or brain magnetic resonance imaging (MRI) abnormalities [110]. In mice, clinical signs appear within the first week of infection and include aberrant locomotion and hind-limb paralysis [113,114]. Demyelination caused by JEV adds to the complications regarding nerve impulse conduction [113].

## 7. Roles of Different ILs

ILs are the proinflammatory cytokines responsible for inflammation via chemically active agents. They are involved in mitogenesis, angiogenesis inhibition, inflammation, chemotaxis, neutrophils degranulation, leukocyte activation, and calcium homeostasis.

### 7.1. IL-6

IL-6 plays a crucial role in altering the permeability of BBB to facilitate JEV entry. Il-6 digests the BBB’s tight junctions leading to CNS inflammation [115,116]. The amount of IL-6 in the CNS rises during JEV infection, but it is inversely related to the quantity of IgM and IgG antibodies. During JEV infection, the amount of IgG varies between primary and secondary viral infection, being higher in the case of secondary infection [117]. Endothelial zonula occludens 1 (ZO1) degradation was aided by soluble bioactive IL-6 produced from JEV-infected pericytes, resulting in barrier disruption [118]. Endothelial changes were accompanied by IL-6-induced Ubiquitin-proteosome-dependent activation mechanism for deterioration [117].

### 7.2. IL-8

IL-8 binds to the G-protein coupled receptors CXCR1 and CXCR2. CXCR2 is also a high-affinity receptor for GRO β, GRO α, GRO γ, and NAP [119]. In a study evaluating the relationship between IL-8, neutrophils, and macrophages, IL-8 content was reported to be higher in the CSF of patients with severe JEV infections than in recovered patients [120]. In the same study, acute JEV patients exhibited higher neutrophil count in CSF, which shows the direct relationship between IL-8 and neutrophils [120]. Another similar study reported the rise of IL-8 and IL-6 in the CNS and peripheral nervous system (PNS) of patients with neurological disorders owing to flavivirus infections [121]. Clearly, the production of IL-8 increases with the increase in the severity of JEV infection.

### 7.3. IL-10

IL-10 plays an important role in the protection of the brain from lethal JEV infection, in addition to regulating immune cell functions. It acts as an immune regulator to protect from tissue damage by an excessive adaptive immune response and proinflammatory mediators. IL-10 amplifies the production of CD8+ T cells, along with energizing B cell differentiation and immunoglobulin secretion. The chief source for the production of IL-10 are thymocytes, B cells, macrophages and keratinocytes. While the T helper 2 (Th2) cells, LY-1+ (CD5+) are responsible for the production of IL-10 in mice, and CD4+ T cells, T cell clones, B cell lymphomas and mast cells are responsible for IL-10 production in humans. In an experiment, the expression of the type II class of IFN (IFN-γ) and signal transducers STAT-1 and STAT-2 were downregulated due to the transfer of JEV immune-splenocyte [50]. JEV-mediated decrease in the generation of IL-10 can be linked with microglial activation and neuronal death [122,123].

### 7.4. Other ILs

JEV’s early neuroinvasion is mediated by IL-1α by interfering with the integrity of the BBB. IL-1α release by JEV-infected peritoneal macrophages has been observed to play crucial roles in augmenting JE-associated pathophysiology in the brain of AXL−/− mice [124]. JEV infection causes microglial activation, which results in the generation of proinflammatory cytokines. HSP60 regulates JEV-induced IL-1β production by activated microglia [125]. JEV infection increases the production of IL-18 and IL-1β in microglia and astrocytes. Replicating JEV activates the inflammasome, which then activates caspase-1 and stimulates the generation of IL-1β and IL-18 [103,126]. IL-1β and IL-18 differentially regulate astrocytes and microglia to release cytokines and chemokines. Interestingly, JEV infection is associated with the suppression of anti-inflammatory cytokine production in the brain. Saxena and colleagues [127] reported that IL-4 and IL-10 expression were both reduced during the progression of JEV infection. IL-4 and IL-10, being anti-inflammatory cytokines, are inversely correlated with neuronal death.

## 8. Therapeutic Insights: Possibilities against JEV Infection in CNS

Despite the fast progress of medical sciences, there is no specific treatment approved for treating JE. Prevention of JEV has achieved some success through vaccination. Structural proteins and NSPs might be promising targets in target-specific treatment approaches. Neuronal receptors can also aid in the same. Several naturally occurring small molecules have exhibited promise against JE.

A multi-target approach to inhibit JEV infection in the population seems a good approach to alleviate JE. Scientists have applied this approach to countering JEV infection by blocking proteins, both structural and non-structural ones, to inhibit the replication machinery. In addition, the blockade of neuronal receptors to inhibit virus entry into the neurons prevents neurological disorders and sequelae [128]. JEV binds to different neuronal receptors, ultimately resulting in neuronal death. Thus, targeting these receptors with viral NSPs participating in replication, such as RdRp, and NS3 protease, would inhibit virus spread and simultaneously stop the cytokine storm [66,129]. This way, JEV-mediated autoimmune disorders would also come to a halt. Active sites for the inhibition of targets in the CNS and viral proteins (Table 3) contain amino acid residues taking part in the binding of ligands for the blockade of these proteins.

Some molecules with reported therapeutic potential against JEV are represented in Table 4. Nature-derived compounds such as arctigenin, a phenylpropanoid dibenzylbutyrolactone lignan, and rosmarinic acid, a phenolic compound, render protection against JEV of the GP78 strain by markedly decreasing JEV-induced neuronal apoptosis, microglial activation, caspase activity and induction of proinflammatory mediators in the brain [142,143]. Kaempferol and baicalin, two flavonoids, demonstrated antiviral activity against JEV. Kaempferol acts by inhibiting the replication of JEV, whereas baicalein exhibits extracellular virucidal activity [144,145]. The United States Food and Drug Administration (FDA)-approved Na^+^/K^+^-ATPase inhibitors, ouabain and digoxin, have displayed antiviral activities through several mechanisms. These glycosides act via inhibiting the Na^+^/K^+^-ATPase pump, leading to alterations in the intracellular concentrations of Na^+^, K^+^, and Ca^2+^, destabilizing the balance of several cellular biosynthetic signaling and vesicular sorting pathways, thus halting JEV infection at the replication stage [146,147]. Ouabain exerts therapeutic effects on JEV infection further by decreasing viral loads and alleviating pathological injuries in the brain, which significantly improves the rate of survival of patients [147]. Ouabain may also block JEV infection by inducing the cellular stress response [148]. Again, the breakdown of BBB by JEV infection might allow otherwise impermeable ouabain to enter into the brain, bind the murine ATPase α2 and α3, and inhibit viral replication in neurons. Mandipine might block the entry, replication, and budding of JEV by downregulating intracellular Ca^2+^ [149]. Genistein and herbimycin A reduce the effect of neurotoxicity induced by JEV and suppress the cachectin (TNF-α) and leukocytic pyrogen (IL-1β) prompted by JEV at the transcriptional level [100,149]. IFN-induced protein with tetratricopeptide repeats 1 (IFIT1) was found to inhibit the replication of JEV by binding to the 5′ -triphosphate RNA and, most preferably, to the 5′ capped 2′-O unmethylated mRNA. NS2A was degraded by tripartite-motif-containing protein 52 (TRIM52) within a proteosome-dependent process through E3 ubiquitin synthetase activity [150]. Overexpression of TRIM52 in BHK-21 cells directly displays E3 Ubiquitin ligase activity and activation of the host’s innate immune system [151]. TRIM52 and nitazoxanide are antiviral compounds reported to possess anti-JEV activity by inhibiting the replication machinery, validated through both in vitro and in vivo methods, suggesting them as potential therapeutic options in the treatment of JE [151,152,153]. A number of nature-derived drugs, namely Kulactone, Nimbolide, gedunin, ohchinin acetate, echinacoside, echinacin, rutin, and cynaroside have been predicted to inhibit RdRp in silico methods, can be validated through in vivo methods [129,154]. JEV expressing GFP reporter gene have been successfully utilized to identify five hit drugs, i.e., lonafarnib, cetylpyridinium chloride, cetrimonium bromide, nitroxoline and hexachlorophene via high throughput screening [128,153].

Several other molecules also exhibit promising potential in modulating different molecular mechanisms. IFN-stimulating genes can be utilized to form an antiviral microenvironment by activating the innate immune response to fight against JEV [158]. Aloe-emodin is well-known for this purpose to induce IFNs [159]. Ribavirin also displays rays of hope by downregulating the synthesis of guanine nucleotides; however, it poses problems regarding non-specificity [160]. Minocycline can reduce oxidative stress by inhibiting the generation of free oxygen radicals [161]. Curcumin can lead to dysregulation of the ubiquitin-proteasome system to minimize the generation of new viral particles [162]. Luteolin can be a potential antiviral drug to treat JEV as it can inhibit the synthesis of E proteins [163]. Apoptozole might be beneficial against JEV since it can hinder the functionality of HSP70 proteins [164]. Viral NS2B-NS3 protease can be inhibited by erythrosine B [165]. Activation and/or upregulation of ERK and MAPK pathways by dehydroepiandrosterone can be highly beneficial in the management of JEV infection [166]. Gene silencing using siRNA holds immense promise against viral infection, viz. domain II of E protein, the coding region of NS5, and the coding sequences of E, M, and NSPs have been successfully silenced experimentally against JEV infection [167]. Again, the E protein domain III binding peptide can be used to inhibit the binding of E protein with the cellular receptor/s [168]. Belladonna acts against JEV by reducing the activities of caspases 3 and 8 and inhibiting the activation of microglia [169]. Amphotericin B has also depicted promise while repurposing by inhibiting the synthesis of viral proteins [170]. Despite so much promise at the preclinical stages, the clinical translation of a safe and effective treatment regimen against JEV is still awaited. A significant issue encountered in many of these strategies is that they were effective when delivered prior to or immediately following infection [171]. In general, it takes a long time for symptoms to appear in a clinical setting, depending on incubation duration, immunological response, and other factors. Treatment is usually initiated after the disease has progressed for some time. As a result, antiviral medicines that are both preventive and therapeutic against JEV are required. Although in vitro and in silico investigations can provide information regarding a drug’s potential antiviral nature as well as evidence of its cytotoxicity profile, a significant disadvantage connected with these study results is the variable translation of the effects in human trials. Attempts to simulate a natural infection in laboratory animals may not predict the intended results in humans accurately.

## 9. Conclusions

Understanding the mechanisms of JEV infection in the CNS is critical for proper management of the same. Certain proinflammatory cytokines and chemokines alter BBB permeability by suppressing the expressions of tight junction proteins, thus making entry of JEV into CNS easier. However, CNS infection begins prior to BBB dysfunction, in line with the characteristics of other flaviviruses. Upregulation of Th1 inflammatory mediators plays havoc in the infection of CNS by JEV. Infected neurons start producing CXCL10, which is further enhanced by the upregulation of IFN-γ. Activated glial cells also produce CXCL10 and CCL5 to promote the migration of monocytes and NK cells into the CNS. Proinflammatory mediators, e.g., IL-6, TNF-α, CCL2, and CCL5, induce irreversible damage to neuronal cells. Interestingly, caveolin-1, GRP78, D2R, TLR7, and Src signaling pathways are important not only during cancer formation but also during JEV-mediated neurodegeneration.

Proinflammatory chemokines and/or cytokines in the CNS, but not JEV itself, promote BBB malfunction during infection. JEV invades within CNS without prior disruption of BBB and infects neurons. Infected neurons, in turn, produce chemokines that can induce the activation of glial cells. In the sequence of things, activated glial cells further upregulate the production of certain cytokines and chemokines. All these mediators act in tandem to reduce the expressions of tight junction proteins in BBB, thereby damaging the integrity of the same. Additionally, these mediators compromise the barrier function by inducing increased expression of adhesion molecules on BBB endothelial cells, allowing for increased infiltration of inflammatory cells from the periphery into CNS. Inflammatory infiltrates can again bring on neuroinflammation and neuronal injury. Current knowledge on how signaling pathways drive the etiology of a panel of neurological disorders is not very clear, in the sense that much remains to be known and decoded in terms of JEV infection signaling pathway components and disease-causal linkages. Further mechanistic understanding of neurological abnormalities will be useful in the creation of tailored therapeutics against JEV infection.

## Figures and Tables

**Table 1:** Classification of eight groups of countries or regions affected by JEV on the basis of their vaccination program.

S. No.	Groups	Rate of Infection and Vaccination Programmes	Countries or Regions	Incidences/100,000	Case Frequency Ratio (Child: Adult)	References
1	A	Vaccination programs of high quality in high-incidence areas	Japan, the Republic of Korea, China Taiwan	0.003	07:01	[20,21,22]
2	B	Areas with extremely low incidence and no immunization programs	Australia, Pakistan, Russia, Singapore	0.003	07:01	[23,24]
3	C	Areas with extremely low incidence and no immunization programs	China	3.3	03:01	[25,26]
4	D	Vaccination programs are weak or non-existent in high-incidence regions.	Cambodia, Indonesia, Laos, Malaysia, Myanmar, Philippines, Timor-Leste	10.6	07:01	[27,28]
5	E	Areas with a medium incidence but no immunization programs	Malaysia, Papua New Guinea	5.3	NA	[29]
6	F	Vaccination programs are being expanded in high-incidence regions.	India, Nepal	2.8	05:04	[22,30]
7	G	Vaccination programs are inadequate or non-existent in low-incidence areas.	Bangladesh, Bhutan, Brunei, Nepal (lower incidence stratum)	1	04:01	[31,32]
8	H	Areas with a medium to a high frequency of disease and growing immunization programs	India (medium incidence stratum), Malaysia (Sarawak), the Republic of Korea, Sri Lanka, Thailand, Vietnam	1.5	07:01	[24,33,34]

**Table 2:** The country or region-wise outbreak, diagnosis, treatment, incidence, and programs run by the government: A global scenario of JE.

Countries or Regions	First Reported	Outbreaks	Diagnosis	Treatments	JE Incidence Rate	Programmes Run by the Governments	References
Australia	1995	2 in 1995, 2 in 1998, and 1 in 2022	Viral antigen detection, JEV-specific antibody detection, reverse passive hemagglutination, staphylococcal co-agglutination tests, ELISA, qPCR, RT-PCR, RT-LAMP	MBDV, Fever relief medicines, plenty of fluid	3 cases	69 million dollar program to control JEV infection, including mosquito control, vaccination and sentinel pig surveillance programs	[35]
Bangladesh	1977	22 patients with 7 deaths in 1977	Viral antigen detection, JEV-specific antibody detection, ELISA, qPCR, RT-PCR	Fever and pain relief medicines, plenty of fluid	0.6–2.7 cases/lakh	-	[9,36]
Bhutan	-	-	ELISA, PCR	Fever and pain relief medicines, fluid	-	Integrated national vaccination program	[8]
Brunei	-	-	ELISA, PCR	Fever and pain relief medicine, plenty of fluid	-	-	[13]
Cambodia	1947	-	ELISA, PCR	LAV-SA14-14-2 vaccine, fever and pain relief medicines, plenty of fluid	11.1 cases/lakh	National vaccination program	[13]
China	1940s	1960–1970 with morbidity >10 cases/lakh	Viral antigen detection, JEV-specific antibody detection, reverse passive hemagglutination, staphylococcal co-agglutination tests, ELISA, qPCR, RT-PCR, RT-LAMP	LAV-SA14-14-2 vaccine, MBDV, fever and pain relief medicines, plenty of fluid	0.1–0.9 cases/lakh	Expanded program on immunization to reduce JE	[37]
Guam	1947	46 reported cases	-	Fever relief medicines, plenty of fluid	-	-	[13]
India	1950	5700 cases with 1315 deaths in 2005	Viral antigen detection, JEV-specific antibody detection, reverse passive hemagglutination, staphylococcal co-agglutination tests, ELISA, qPCR, RT-PCR, RT-LAMP	LAV-SA14-14-2 vaccine, fever and pain relief medicines, plenty of fluid	15 cases/lakh	Government immunization program	[38]
Indonesia	1974	-	JEV-specific antibody detection, ELISA, PCR, RT-PCR	Fever and pain relief medicines, plenty of fluid	8.2 cases/lakh	No vaccination program	[9,39]
Japan	1933	Mainly before 1960	Viral antigen detection, JEV-specific antibody detection, reverse passive hemagglutination, staphylococcal co-agglutination tests, ELISA, qPCR, RT-PCR	VCDV-Bejing-I vaccine, pain and fever relief medicines	<10 cases	Vaccination program	[9]
Laos	1989	-	JEV-specific antibody detection in the CSF	Fever and pain relief medicines, fluids	-	JEV vaccination in 2013	[40]
Malaysia	1952	154 cases with 42 deaths in 1999	Viral antigen detection, JEV-specific antibody detection, ELISA, qPCR	MBDV, fever relief medicines	4.3 cases/lakh	JE vaccination was introduced in July 2001, and the vaccination was only practiced in Sarawak and formalin-activated mouse-derived JE vaccine (Biken, Japan) is used in Malaysia	[41]
Myanmar	1968	5 cases with 4 deaths in 1947 and 43 with 32 deaths in 1948	Viral antigen detection, JEV-specific antibody detection, ELISA, qPCR, RT-PCR	Fever and pain relief medicines, plenty of fluid	-	National vaccination program	[9,13]
Nepal	1978	2040 cases with 205 deaths in 2005	JEV-specific antibody detection, ELISA	LASV-SA14-14-2 vaccine, fever relief medicines	1.3 cases/lakh	National JE prevention and control program in Kathmandu valley	[31]
Pakistan	1980s	-	JEV-specific antibody detection	Fever relief medicines	-	No vaccination programs	[9]
Papua New Guinea	1995	-	JEV-specific antibody detection, ELISA, PCR	Fever relief medicines	-	-	[42]
Philippines	1950s	-	-	-	-	-	[13]
Saipan, USA	1990	1990	JEV-specific antibody detection in CSF, ELISA	Fever relief medicines	-	no vaccination programs	[43]
Singapore	1952	-	Viral antigen detection, JEV-specific antibody detection, reverse passive hemagglutination, staphylococcal co-agglutination tests, ELISA, qPCR, RT-PCR	Fever relief medicines	<5 cases	Vaccination programs reduce JE case	[9,13]
Republic of Korea	1946	-	JEV-specific antibody detection in serum and CSF, ELISA, PRNT, PCR	MBDV, fever relief medicines	0.01–0.08 cases/lakh and <10 cases/year	Successful vaccination program for three decades in the Republic of Korea	[44]
Sri Lanka	1968	-	ELISA, PCR	Fever relief medicines	<100 cases	JE vaccination program	[9,13]
China Taiwan	1938	1960–1970 with morbidity ~12.4 cases/lakh	Viral antigen detection, JEV-specific antibody detection, ELISA, qPCR, RT-PCR	MBDV, Fever relief medicines, plenty of fluid	0.03/lakh	Taiwan National Infectious Disease Statistics System–Japanese Encephalitis, Self-reporting through the toll-free 1922 hotline or local public health authority.	[45]
Thailand	1961	-	RT-PCR	MBD vaccine, fever relief medicines	300 cases/year	vaccination program	[9,13]
Vietnam	1960	-	-	MBDV, fever relief medicines	1–8 cases/lakh	vaccination program (from 1997)	[9,13]

CSF, cerebrospinal fluid; ELISA, enzyme-linked immunoassay; PCR, polymerase chain reaction; PRNT, plaque reduction neutralization test; qPCR, quantitative PCR; RT-LAMP, reverse transcription loop-mediated isothermal amplification, RT-PCR, reverse transcription PCR.

**Table 3:** In vitro, in vivo, and in silico targets for inhibiting JEV infection, targeting replication, host binding, autoimmunity, and cytokine generation, together with their active pocket residues and native ligands from crystal structures.

S. No.	Name of Targets	PDB ID	Ligands	Amino Acid Residues of Active Sites	Resolution of Crystal Structures	References
1	CLEC5A	2YHF	NA	-	1.9 Å	[130]
2	GRP78	5F1X	ATP	Thr37, Thr38, Tyr39, Gly227, Gly228, Thr229, Glu293, Lys296, Ser300, Gly364,	1.9 Å	[131]
3	TLR7	6LW1	RIJUCCOLHSAZPO-GOTSBHOMSA-N	Asn265, Tyr264, Phe349, Glu352, Leu353, Gln354, Val355, Val381, Thr406, Phe408, Phe507, Ser530, Gln531	2.8 Å	[132]
4	D2R	7DFP	DKGZKTPJOSAWFA-UHFFFAOYSA-N	Val91, Leu94, Val111, Asp114, Val115, Cys118, Cys182, Ile184, Trp386, Phe390, Thr412, Tyr416,	3.1 Å	[133]
5	Src	1FBZ	SPSGYTWOIGAABK-DQEYMECFSA-N	Arg12, Arg32, Glu35, Ser36, His58, Lys60	2.4 Å	[134]
6	Caveolin-1	7SC0	NA	-	3.4 Å	[135]
7	Capsid	5OW2	KRKNYBCHXYNGOX-UHFFFAOYSA-K	Pro43, Val44,	1.98 Å	[136]
8	Envelop	5MV1	NA	-	2.25 Å	[137]
9	NS1	5O36	QAOWNCQODCNURD-UHFFFAOYSA-L	Arg347, Gln349	2.6 Å	[138]
10	NS3	2Z83	NA	-	-	[139]
11	NS5(RdRp)	4HDH	ZKHQWZAMYRWXGA-KQYNXXCUSA-N	Arg460, Arg474, Asp668, Ser715, Arg734, Arg742, Ser799, Trp800,	2.28 Å	[140]
12	NS3/NS4A	5WX1	NA	-	2.35 Å	[140]
13	NS2b-NS3	4R8T	VEXZGXHMUGYJMC-UHFFFAOYSA-M	Gly151	2.133 Å	[141]

**Table 4:** Preclinically validated molecules inhibiting JEV infections.

S. No.	Nature-Derived Compounds	Mechanisms	References
1	Arctigenin	Decreases JEV-induced neuronal apoptosis, microglial activation, and caspase activity.	[142]
2	Baicalein	Extracellular virucidal activity.	[145]
3	Cilnidipine	Inhibits JEV in high-throughput screening assay (HTS) with EC_50_ of 6.52 µM.	[155]
4	Cinaroside	Inhibits non-structural protein (RdRp) in silico.	[129]
5	Digoxin	Reported to act as an inhibitor of the Na^+^/K^+^-ATPase pump.	[146]
6	Echinacin	Inhibits RdRp in silico.	[129]
7	Echinacoside	Inhibits RdRp in silico.	[129]
8	FGIM-1-27	Inhibits JEV in high-throughput screening assay (HTS) with EC50 s of 3.21 µM.	[155]
9	Gedunin	Inhibits RdRp in silico.	[154]
10	Genistein	Reduces the effect of neurotoxicity induced by JEV and suppresses the cachectin (TNF-α) and leukocytic pyrogen (IL-1β) prompted by JEV at the transcriptional level.	[149]
11	Herbimycin A	Reduces the effect of neurotoxicity induced by JEV and suppresses the cachectin (TNF-α) and leukocytic pyrogen (IL-1β) prompted by JEV at the transcriptional level.	[149]
12	IFIT 1	Inhibits JEV replication by binding to the 5′ -triphosphate RNA and, most preferably, to the 5′ capped 2′-O unmethylated mRNA.	[150]
13	Kaempferol-3-glucoside	Inhibits RdRp in silico.	[129]
14	Kulactone	Inhibits RdRp in silico.	[154]
15	Manidipine	Inhibits intracellular Ca^2+^, which is required for JEV entry, replication, and budding.	[149]
16	Mycophenolic acid	Reported antiviral activity of an immune suppressant as an anti-JEV drug via plaque reduction neutralization assay, virus yield reduction assay, and cytopathic effect inhibition assay, accompanied by an IC_50_ of 3.1 µg/mL through in vivo and in vitro experiments.	[156]
17	Niclosamide	Inhibits JEV with EC_50_ of 5.80 µM	[155]
18	Nimbolide	Inhibits RdRp in silico.	[154]
19	Nitazoxanide	Inhibits the replication machinery, validated through both in vivo as well as in vitro methods, which suggests this compound is a potential agent for JE treatment.	[152,157]
20	Ohchinin acetate	Inhibits non-structural protein (RdRp) in silico.	[154]
21	Ouabain	Reported against the Na^+^/K^+^-ATPase as an inhibitor during the replication of the JEV in the BALB/C mouse model	[147]
22	PP2	Reduces the effect of neurotoxicity induced by JEV and suppresses the Cachectin (TNF-α) and leukocytic pyrogen (IL-1β) prompted by JEV at the transcriptional level.	[149]
23	Quercetagetin 7-glucoside	Inhibits RdRp in silico.	[129]
24	Rosmarinic acid	Reduces induction of proinflammatory mediators, neuronal apoptosis, microglial activation, and caspase activation.	[143]
25	Rutin	Inhibits RdRp in silico.	[129]
26	TRIM52	NS2A was degraded by TRIM 52 within a proteosome-dependent process through E3 Ubiquitin synthetase activity. Overexpression of TRIM52 in BHK-21 cells directly shows E3 Ubiquitin ligase activity and activation of the host innate immune system.	[151]

## Data Availability

Not applicable.
